# The Effect of Milling on the Ethanolic Extract Composition of Dried Walnut (*Juglans regia* L.) Shells

**DOI:** 10.3390/ijms241713059

**Published:** 2023-08-22

**Authors:** Giovanni Ventura, Davide Mesto, Davide Blasi, Tommaso R. I. Cataldi, Cosima Damiana Calvano

**Affiliations:** 1 Department of Chemistry, University of Bari Aldo Moro, Via Orabona 4, 70126 Bari, Italy; davide.mesto@uniba.it (D.M.); tommaso.cataldi@uniba.it (T.R.I.C.); cosimadamiana.calvano@uniba.it (C.D.C.); 2 Interdepartmental Research Center SMART, University of Bari Aldo Moro, Via Orabona 4, 70126 Bari, Italy

**Keywords:** biomass, epoxidation, fatty acids, LC-MS/MS, mill grinding, phenols, walnut shells

## Abstract

This study investigates the ethanolic extract of dried walnut (*Juglans regia* L.) shells upon hammer milling (HM) and ball milling (BM) grinding processes. Marked differences were observed in the attenuated total reflection Fourier-transform infrared (ATR-FTIR) spectra. The two extracts were investigated by reversed-phase liquid chromatography coupled with electrospray ionization and high-resolution mass spectrometry (RPLC-ESI-HRMS). Following enzymatic digestion, the fatty acids (FAs) were examined, and tandem MS of epoxidized species was applied to establish the C-C double bond position; the most abundant species were FA 18:2 Δ^9,12^, FA 18:1 Δ^9^, and FA 18:3 Δ^9,12,15^. However, no significant qualitative differences were observed between FAs in the two samples. Thus, the presence of potential active secondary metabolites was explored, and more than 30 phenolic compounds, including phenols, ellagic acid derivatives, and flavonoids, were found. Interestingly, the HM samples showed a high concentration of ellagitannins and hydrolyzable tannins, which were absent in the BM sample. These findings corroborate the greater phenolic content in the HM sample, as evaluated by the Folin–Ciocalteu test. Among the others, the occurrence of lanceoloside A at *m*/*z* 391.1037 [C_19_H_20_O_9_-H]^−^, and a closely related benzoyl derivate at *m*/*z* 405.1190 (C_20_H_22_O_9_-H]^−^), was ascertained. The study provides valuable information that highlights the significance of physical pre-treatments, such as mill grinding, in shaping the composition of extracts, with potential applications in the biorefinery or pharmaceutical industries.

## 1. Introduction

The agri-food industry generates billions of tons of waste annually, consisting of non-edible portions of fruits and vegetables such as husks, peels, and shells. These residues not only contribute to environmental pollution but also pose severe managerial and economic problems for the industry [1]. Walnut (*Juglans regia* L.) is one of the world’s high demand “dried fruits”, along with almonds, cashews, and hazelnuts. They are largely consumed thanks to the pleasant taste and the numerous nutritional, health, and sensory features [2].

Global walnut production has steadily grown, reaching an impressive 2.6 million metric tons in the 2022/23 season (https://www.statista.com/statistics/675967/walnut-production-worldwide, accessed on 1 August 2023). Upon kernel extraction, the shell, which represents more than half of the total weight of the whole walnut, becomes an agro-industrial waste, and it is often burned as fuel. To reduce the environmental impact and provide added value to this by-product, various alternative applications for walnut shells have been recently proposed. These include using them as a strengthening filler in polymers [3], as a bioremediation agent for the removal of cephalexin [4], heavy metals [5], volatile organic compounds, and dyes [6] from aqueous solution, and as a material for the synthesis of porous carbon electrodes [7]. 

Since approximately 50% of the dried shell mass are aromatic biopolymers, walnut shells have been recently used as a promising feedstock for the fractionation of organosolv lignin [8,9]. Before lignin extraction, extractives are usually removed because they would contaminate the isolated lignin fractions. In the perspective of a zero-waste biorefinery process, in which pure extractives, lignin, hemicellulose, and cellulose are fractionated and valorized, the definition of the global composition plays a key role in determining the economic sustainability of the process. Extracts from walnut shells are a precious source of bioactive compounds with remarkable antioxidant activity [10,11] and potential applications spacing from cosmetics to nutraceuticals. In this context, some of us recently reported on the lignin ethanosolv fractionation from dried walnut shells focusing on the role of biomass pre-treatment conditions [12]. Extractives were isolated using a Soxhlet apparatus and food-grade ethanol as a solvent. The radical scavenger activity (RSA) of extracts was strongly affected by the feedstock pre-treatment conditions. Specifically, extracts obtained from the ball-milled (BM) biomass showed an inhibition concentration (IC_50_) three orders of magnitude lower than those obtained by coarse fragmentation, i.e., hammer- milled (HM) biomass. This work aims to move one step forward in understanding the impact of biomass grinding on the extract composition of walnut shells and their valorisation, starting from the low molecular weight fractions.

Several studies [13,14,15] have reported the positive health effects of walnut kernels or walnut oil [14], which are rich in nutrients such as vitamins, amino acids, carbohydrates, dietary fibre, minerals, and lipids, but very few studies analyzed the composition of the shell in terms of bioactive compounds [16,17], and how this composition varies depending on the processing conditions. Fatty acids are beneficial for human health and can be employed for biofuel synthesis. Walnut kernels show the lowest ratio of saturated fatty acids (SFAs) to total fatty acids and a larger percentage of polyunsaturated fatty acids (PUFAs) [18] compared to most other nuts. Regardless of the investigated sample, the fatty acid profile is typically obtained using gas chromatography (GC) [18]. However, the sample must be crushed, and oil must be extracted, subjected to a trans-esterification with a methanolic potassium hydroxide solution, and the generated fatty acid methyl esters (FAMEs) separated in the GC column [19]. This protocol may involve the use of contaminant reagents, and it needs a considerable amount of time and other resources with a possible global sample loss. In the present study, a method for the analysis of the total fatty acids (TFAs) by reversed-phase (RP) liquid chromatography coupled with a high-resolution (HR) mass spectrometry (MS) system is developed. Additionally, the position of the double bonds in unsaturated FAs was established by an epoxidation reaction using *meta*-chloroperoxybenzoic acid (*m*-CPBA) [20,21], followed by MS/MS analyses. 

By using RPLC-ESI-MS/MS but different chromatographic conditions, phenolic compounds were also characterized, including phenols, ellagic acid derivatives, flavonoids, and ellagitannins. Interestingly, the composition’s comparison between grinding methods revealed significant differences in terms of potentially interesting compounds, such as hydrolyzable tannins. This study sheds light on the significance of the biomass pre-treatment problem in identifying conditions for the highest recovery of desired bioactive substances.

## 2. Results and Discussions

### 2.1. ATR-FTIR Characterization of Ball Milling Ethanolic Extracts of Walnut Shells

This study starts from the evidence of the enormous difference in the radical scavenger activity (RSA) of hammer-milled (HM) compared to ball-milled (BM) dried walnut shell extracts, as determined via 2,2-diphenyl-1-picrylhydrazyl (DPPH) colorimetric assay. Indeed, the HM extract exhibited an inhibition concentration (IC_50_) of 0.235 ± 0.005 mg/mL that drastically increased up to 116 ± 3 mg/mL in the case of the BM samples [12], thus indicating a higher RSA in the less powdered HM sample. 

**Figure 1:**
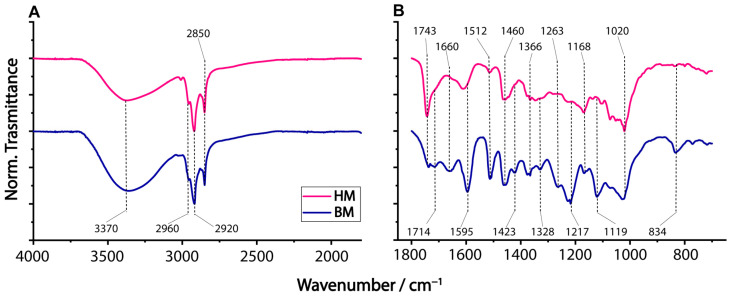
ATR-IR spectra of walnut (*Juglans regia* L.) shell extracts obtained by hammer-milled (HM, pink line) and ball-milled (BM, blue line) biomass in the region (**A**) 4000–1800 cm^−1^ and (**B**) 1800–600 cm^−1^.

A qualitative idea of the different composition of BM and HM dried walnut extracts can be appreciated by their ATR-FTIR spectral comparison. Plots A and B of Figure 1 display the normalized ATR-FTIR spectra in the region 4000–1800 cm^−1^ and 1800–600 cm^−1^, respectively. 

In both spectra, the most intense signals are those due to methyl and methylene groups (2960, 2920, 2850, and 1460 cm^−1^). The hydroxyl group, which peaked at 3370 cm^−1^, was relatively more intense in the BM sample. The C=O stretching band of unconjugated ketones and esters showed an intense peak at 1743 cm^−1^ and a shoulder at 1714 cm^−1^ (aromatic acids) [22] in the HM sample, while both signals exhibited roughly the same intensity in the BM one. The observed signal at 1660 cm^−1^ was likely due to conjugated C=O [23] or C=C stretching of non-aromatic alkenes. In general, the ATR-IR spectrum of BM indicates the occurrence of high amounts of aromatics. Indeed, the peak signals at 1595, 1328, and 834 cm^−1^ are typical of syringyl units [22,23]; those at 1512, 1263, and 1217 cm^−1^ are due to guaiacyl ones [24], which may be related to the partial lignin depolymerization [12]. Signals of phenolic -OH can be seen at 1366 and 1328 cm^−1^ [24], while signals in the region 900–1100 cm^−1^ are due to the presence of carbohydrates [25]. 

These differences could be potentially ascribed to changes in the free content of phenolic components and unsaturated fatty acids (UFAs), which will be discussed in the following paragraphs.

### 2.2. Separation and Detection of Fatty Acids by RPLC-ESI-MS

The total fatty acid (TFA) profile of dried powdered walnut shell extracts was obtained by enzymatic hydrolysis using *Candida rugosa* lipase. This enzyme catalyzes the hydrolysis of triglycerides, releasing bound fatty acyl chains. Each digested lipid extract was examined by RPLC coupled with Fourier-transform mass spectrometry (FTMS) via electrospray ionization (ESI) in negative-ion mode ([M-H]^−^) to exploit the existence of a carboxylic functional group in each FA [26]. A thorough analysis of FAs implies the identification of the C-C double bond location in unsaturated fatty acyl chains. To address this challenge, we used an oxidation reaction [21] that produces an epoxidized fatty acid (epo-FA) whose MS/MS spectrum allowed us to unambiguously obtain this information.

**Figure 2:**
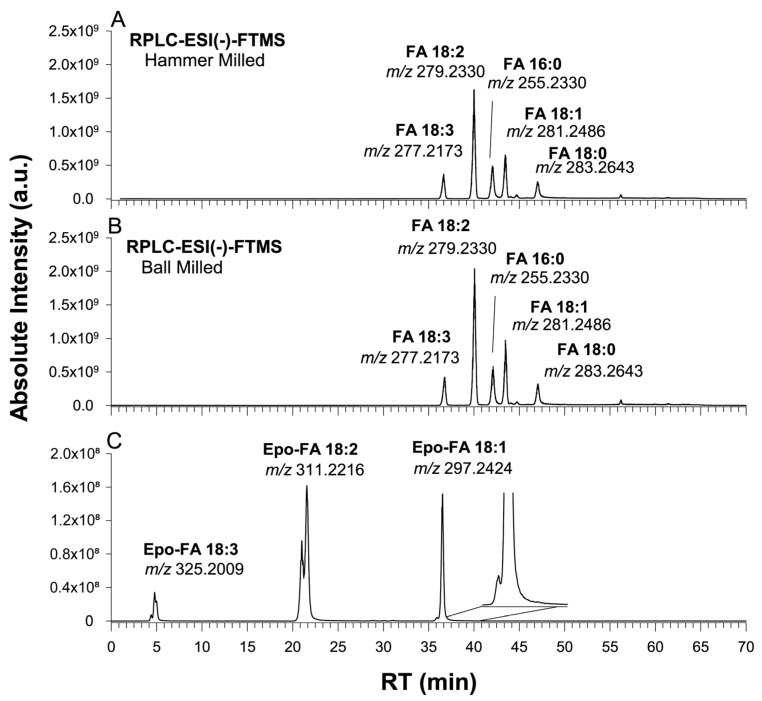
Multiple EIC chromatograms by RPLC-ESI-MS in the negative-ion mode of FAs occurring in the ethanolic extracts of dried walnut shells of (**A**) HM and (**B**) BM samples. (**C**) EIC chromatogram after the epoxidation reaction of a BM extract with *m*-CPBA.

The settled chromatographic conditions led to the separation of FAs based on their acyl chain length and the number of double bonds, as demonstrated in plots A and B of Figure 2 for HM and BM samples, respectively.

The identification of each FA was successfully obtained by accurate *m*/*z* values retrieved from the full mass spectra, using the online lipid database (www.alex123.info, accessed on 15 June 2023) and setting a mass accuracy of ≤3 ppm. As an output, the total number of carbon atoms and unsaturations (annotated as C:DB [27]) was reported. The matching of retention time data with previous works [28,29] confirmed the assignment listed in Table 1. As expected, the retention of FAs on a reversed-phase column increases as the number of carbon atoms on the acyl chain rises. Species with the same number of carbon atoms and more unsaturations elute sooner, as they are relatively more polar and interact less with the hydrophobic stationary phase. As can be seen, no significant differences in plots A and B were observed. The relative abundance of FAs was assessed by calculating the chromatographic peak area of each FA and dividing it by the sum areas extended to all detected FAs. In the analyzed samples, UFAs with 18 carbon atoms were found to be the most abundant species. Specifically, in both HM and BM samples, the FA 18:2 accounted for nearly 40%, followed by FA 18:1 (16–18%) and FA 18:3 (approximately 10%). Among the SFAs, FA 16:0 and FA 18:0 were the most abundant, ranging from 14–15% to 9–10%, respectively. The relative abundances of both samples are reported in Table 1. Note that the FA composition evaluated in the powdered walnut shells mirrors the distribution commonly seen in kernel samples [18], making it appealing enough to recover these compounds from by-products as an added value. Though an excess of SFAs is detrimental to the cardiovascular system [30], a high intake of polyunsaturated fatty acids (PUFAs), especially ω-3 fatty acids, positively affects human health [31]. In the present study, it is confirmed that walnut shells are a good source of mono- and polyunsaturated fatty acids. Given the widespread worldwide distribution of these fruits, walnut shells are an excellent candidate for extracting these compounds and exploiting the positive health effects.

### 2.3. Double Bond Position by Tandem MS of Epoxidized FAs

**Figure 3:**
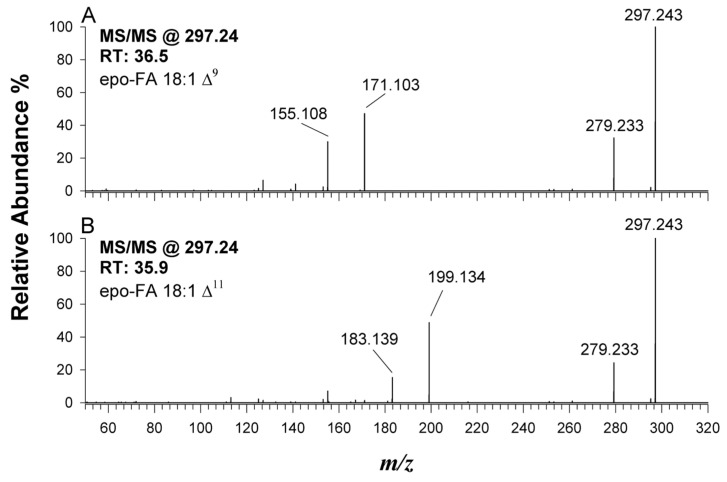
ESI(−)-HCD-MS/MS product ion spectra of deprotonated epo-FA 18:1 at *m*/*z* 297.23, corresponding to two isomers free FAs eluting at (**A**) 36.5 and (**B**) 35.9 min, respectively. A total of 30% of normalized collision energy (NCE) was applied.

Structural information in terms of double bonds location on UFAs was obtained by RPLC-ESI(−)-FTMS/MS analyses of the corresponding epo-FAs in the higher-energy collisional dissociation (HCD) regime. The profile shown in Figure 2C represents the extracted ion current (EIC) chromatogram obtained on the digested lipid extract after the epoxidation reaction considering a complete conversion of all the possible double bonds, as we previously demonstrated on other compounds [20,21]. As emphasized in the inset of Figure 2C, the EIC chromatogram of epo-FA 18:1 exhibited a highly intense peak at 36.5 min and a barely discernible one at a slightly lower retention time (RT) of 35.9 min. As reported in Figure 3, the DB location was assigned to each epoFA 18:1 by tandem MS. 

According to Feng et al. [32], in the MS/MS spectrum of an epo-FA, a couple of diagnostic ions with a mass difference of 15.995 Da due to the formation of a deprotonated aldehyde and an olefin species arises from the cleavage of the epoxide ring. The *m*/*z* ratio of these product ions depends on the epoxide location, which is strictly correlated to the DB position along the fatty acyl chain. For instance, both product ions at *m*/*z* 155.108 and 171.103 observed in Figure 3A (precursor ion at *m*/*z* 297.2429 with RT 36.5 min) correspond to the diagnostic ion fragments of the constitutional isomer ∆^9^ (i.e., oleic acid, most likely), while the couple at *m*/*z* 183.139/199.134 (plot B) provided evidence for the occurrence of small amounts of the isomer ∆^11^ (i.e., possibly elaidic acid), thus confirming the epoxidation process ideally suited for the analysis of monounsaturated fatty chains.

Following the same rationale, the DB position of other UFAs was established. When epoxidized PUFAs are examined by HCD-MS/MS, additional fragmentation processes occur, leading to the formation of collateral ions, which can make it challenging to determine the C-C double bond position. Nevertheless, the presence of one doubly unsaturated FA and its accurate *m*/*z* values allow us to confidently identify the product ions that indicate the position of the first double bond.

Indeed, the MS/MS of epo^2^-FA 18:2 gives rise to product ions at *m*/*z* 155.108/171.103 and *m*/*z* 183.139/199.134, suggesting the presence of two constitutional isomers bearing the first C=C from carboxylic moiety on the carbon atom 9 or 11, while the observed signals at *m*/*z* 211.134/227.129 and *m*/*z* 193.123/209.118 provide evidence of a second double bond at the ∆^12^ location (i.e., ∆^9,12^). Despite the absence of diagnostic ions, the minor isomer was putatively identified as ∆^11,14^ since double bonds are usually not conjugated in FAs.

Finally, the MS/MS spectrum of an epo^3^-FA 18:3 exhibits a more complex profile, but very recently [20], the same behavior was observed with anacardic acids. Therefore, the peak signals of the first and second double bond positions, namely ∆^9,12^, were still recognized. The product ions at *m*/*z* 249.149 and *m*/*z* 267.160 revealed the presence of an additional C-C double bond at ∆^15^. Note that the increased hydrophilicity of the epoxidation reaction products results in earlier elution, and the possible stereoisomers ensuing from the epoxidation reaction can contribute to peak broadening. Although the presence of other UFAs cannot be ruled out, the identification of FA 18:3 ∆^9,12,15^ as the major component is plausible. All assignments are summarized in Table 1.

### 2.4. LC-MS Characterization of Phenolic Compounds in Walnut Shell Extracts

RPLC-ESI-FTMS was also used to investigate the phenolic compounds of both dried walnut ethanol extracts obtained by HM and BM grinding, as fatty acids showed only a small variability between the two samples. The choice of ESI in negative-ion mode was based on the remarkable ionization efficiency of the compounds of interest, chiefly attributed to the presence of carboxylic and/or hydroxyl groups [33]. The accurate *m*/*z* ratios obtained using the Orbitrap analyzer alongside the acquisition of tandem mass spectra enabled a comprehensive characterization of the ethanolic walnut shell extracts.

**Figure 4:**
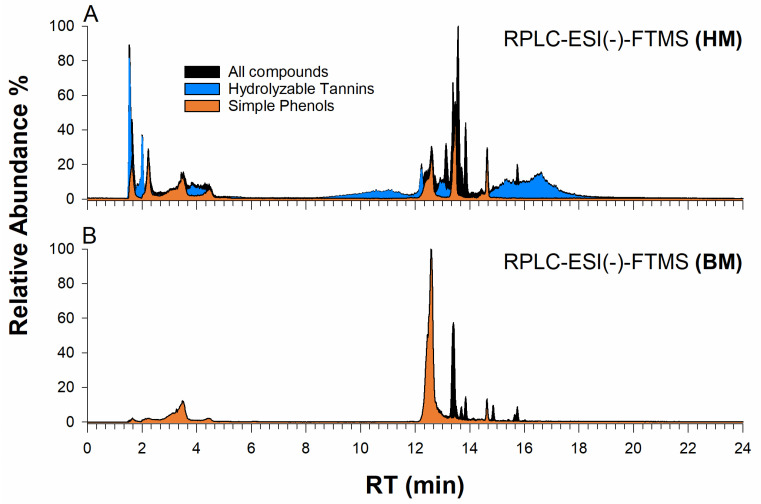
Multiple EIC chromatograms by RPLC-ESI-MS in the negative-ion mode of phenolic compounds occurring in (**A**) HM and (**B**) BM ethanolic extracts of walnut shells (black profiles). The EIC chromatograms of hydrolyzable tannins and simple phenols are shown in blue and orange, respectively.

Firstly, the analyses focused on the HM sample due to the putatively higher RSA [12]. The walnut shell extracts demonstrated a complex profile comprising hydrolyzable tannins, flavonoids, and phenolic compounds. Up to thirty compounds were identified and listed in Table 2, providing accurate *m*/*z* values, three diagnostic ions in HCD tandem MS spectra, classification, and relative abundance in both HM and BM analyzed samples. The multi-EIC chromatogram of chosen compounds is presented in Figure 4A. 

Among the identified compounds, phenolic acids, including benzoic, hydroxybenzoic, protocatechuic, and gallic acids, played a prominent role. Phenolic acids are a class of compounds commonly found in plants with important antioxidant properties, often associated with various health benefits [34,35]. Gallic acid, for instance, is known for its potent antioxidant and anticancer properties [36,37], while protocatechuic acid has been studied for its anti-inflammatory and hepatoprotective effects [38]. In their MS/MS spectra, the deprotonated molecule [M-H]^−^ of these compounds exhibited minimal fragmentation, with only a diagnostic ion at [M-43.990]^−^ being observed, which can be easily ascribed to the loss of CO_2_. Interestingly, gallic acid (3,4,5-trihydroxy benzoic acid, C_7_H_6_O_5_, monoisotopic mass 170.0215) and protocatechuic acid (3,4-dihydroxybenzoic acid, C_7_H_6_O_4_, 154.0266) were also detected as ethylated species at *m*/*z* 197.0457 and *m*/*z* 181.0500, which are likely formed during the extraction process. Their MS/MS spectra were easily recognizable, showing a neutral loss of ethylene, 28.031 Da, followed by the typical loss of CO_2_ (not shown). The chromatographic profiles of walnut shell ethanolic extracts revealed the abundant occurrence of ellagic acid (2,3,7,8-tetrahydroxy [1]benzopyrano [5,4,3-cde][1]benzopyran-5,10-dione, C_14_H_6_O_8_, 302,0063 Da) and its derivatives, such as pentoside and hexoside compounds well-known for their antioxidant properties and health benefits [39,40]. Ellagic acid is particularly abundant in walnut kernels and shells, making them one of the richest sources of this compound among nuts. Due to its structural rigidity, the MS/MS spectrum of ellagic acid shows signals primarily attributed to the loss of CO and CO_2_, albeit with low intensity.

**Figure 5:**
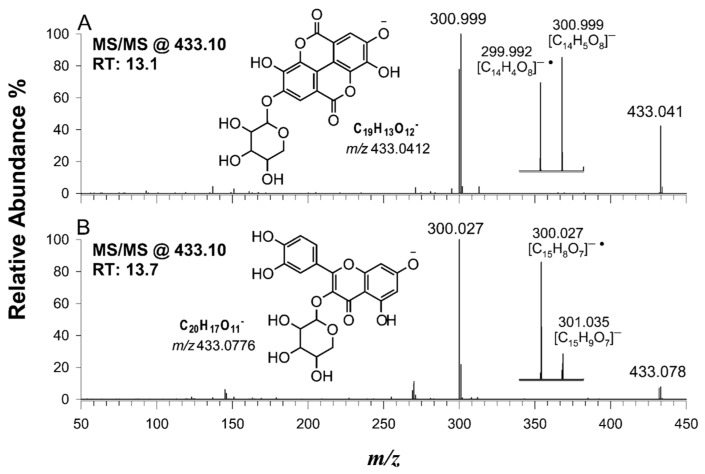
HCD-MS/MS spectra of precursor ions at *m*/*z* 433.10 due to (**A**) an ellagic acid pentoside and (**B**) a quercetin pentoside (see compounds 18 and 25 in Table 2). Putative structures are reported in the insets, along with the enlargements of the ions generated upon detachment of the pentose moiety with the formation of typical peak doublets. A total of 30% of normalized collision energy (NCE) was applied.

Figure 5 shows the MS/MS spectra of putatively identified ellagic acid pentoside and quercetin pentoside. The monoisotopic masses of these two deprotonated compounds differ by 0.036 *m*/*z* units (8.3 ppm), and they are chromatographically separated. During the fragmentation process, both compounds experienced the loss of a pentoside moiety, giving rise to a typical doublet where both the anion and the radical anion were observed. The FTMS instrument enabled the unambiguous identification of a single possible empirical formula within a 5-ppm accuracy, as depicted in the insets of Figure 5. The MS/MS spectrum of ellagic acid pentoside is shown in Figure 5A.

Ellagic acid derivatives with a sugar moiety are easily recognizable, as their MS/MS spectra exhibit a characteristic doublet of signals at *m*/*z* 300 and 301. Such a doublet is a marker of sugar loss as a neutral or radical fragment, leading to the formation of ellagic acid anion and radical anion, respectively. This unusual fragmentation behavior can be explained thanks to the ability to stabilize the unpaired electron in a highly conjugated system (vide infra).

Ellagitannins, derived from the secondary metabolism of dicotyledonous *Angiosperms* plants [41], represent one of the main classes of polyphenols. Their fundamental chemical structure consists of a central carbohydrate core, typically D-glucopyranose, linked to various units of 3,4,5-trihydroxy benzoic acid through ester bonds. These gallic acid units can further connect through C-C and C-O biaryl bonds, resulting from both inter- and intra-molecular phenolic oxidative coupling processes, exemplified in the hexa-hydroxydiphenoyl (HHDP) unit. Following ingestion, ellagitannins undergo hydrolysis, releasing ellagic acid [42,43,44]; ellagitannins possess remarkable antioxidant and anti-inflammatory properties, with numerous studies indicating their potential role in contrasting diseases such as cancer and cardiovascular and neurodegenerative disorders [45]. The walnut shell extracts exhibited the presence of various gallotannins, including monogalloyl-hexose, galloyl-HHDP-hexose, bis-HHDP-hexose, digalloyl-HHDP-hexose, digalloyl-bis-HHDP-hexose, and trigalloyl-HHDP-hexose, with D-glucopyranose being the most common hexose moiety. Due to the presence of gallic acid or HDDP in the chemical structure of these compounds, the ESI(-) MS/MS spectra often exhibited intense signals at *m*/*z* 169 and 301 or signals resulting from neutral losses of these moieties.

Moreover, the presence of flavonoids, such as quercetin pentoside, quercitrin, and quercetin-hexoside, further enhances the extract’s bioactivity; flavonoids are renowned for their potent antioxidant and anti-inflammatory properties [46,47,48]. An orbitrap analyzer enables effective discrimination of flavonoids from isobaric ellagic acid derivatives, providing valuable insights into the phenolic compounds occurring in the walnut shell extract.

**Figure 6:**
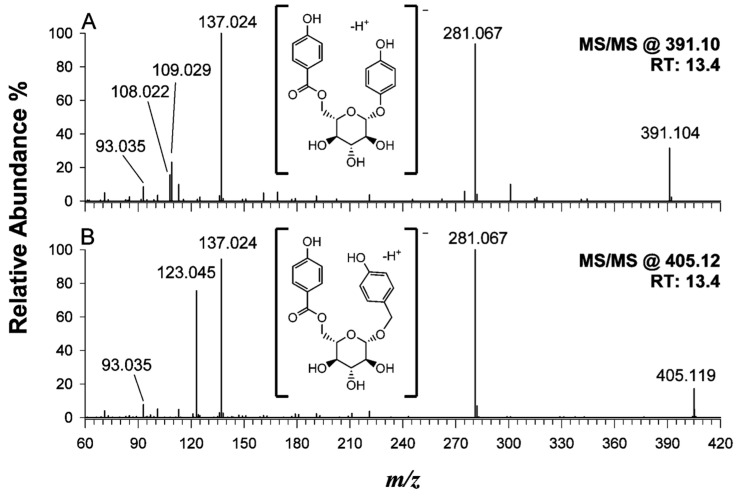
Tandem MS spectra of precursor ions at (**A**) *m*/*z* 391.10 and (**B**) *m*/*z* 405.12, due to compounds 19 and 20 reported in Table 2, putatively identified as lanceoloside A (i.e., 4-hydroxyphenyl 6-O-(4-hydroxybenzoyl)-β-D-glucopyranoside and a 4-hydroxybenzyl derivate of lanceoloside A (e.g., 4-hydroxybenzyl-6-*O*-(4-hydroxybenzoyl)-β-*D*-glucopyranoside). Note that in spectrum A, the product ion at *m*/*z* 109.029 is accompanied by the formation of a radical anion at *m*/*z* 108.022 with the chemical formula [C_6_H_4_O_2_]^•−^ stabilized by resonance. NCE: 30%.

Finally, the putative identification of two coeluting phenolic glycosides (19 and 20 in Table 2) at 13.4 min is described. Their MS/MS spectra at *m*/*z* 391.10 and 405.12 are depicted in Figure 6, plots A and B, respectively.

Both spectra exhibited common product ions at *m*/*z* 281.024 and *m*/*z* 137.067, recognized as deprotonated hydroxybenzoic acid and hydroxybenzoic acid conjugated to a hexosyl group. Moreover, in the spectrum reported in plot A, two diagnostic signals at *m*/*z* 109.029 and *m*/*z* 108.022 are present, implying the occurrence of a dihydroxybenzene radical anion with the chemical formula [C_6_H_4_O_2_]^•−^ stabilized by resonance. The tandem MS spectrum of phenolic 20 at *m*/*z* 405.12 (see plot B of Figure 6) exhibited a peak signal at *m*/*z* 123.045, suggesting the presence of an additional methylene moiety. While further experiments and the use of standards are mandatory to definitively identify these benzoylglucosides, the plausible identification of lanceoloside A (i.e., 4-hydroxyphenyl 6-O-(4-hydroxybenzoyl)-β-D-glucopyranoside) as a deprotonated molecule at *m*/*z* 391.1037 (see the suggested structure in inset), also known as breynioside A (C_19_H_20_O_9_, monoisotopic mass 392.1107 Da) and a 4-hydroxybenzyl derivate of lanceoloside A (i.e., 4-hydroxybenzyl-6-O-(4-hydroxybenzoyl)-hexoside) at *m*/*z* 405.1190 (C_20_H_22_O_9_, monoisotopic mass 406.1264 Da) is proposed, respectively.

**Figure 7:**
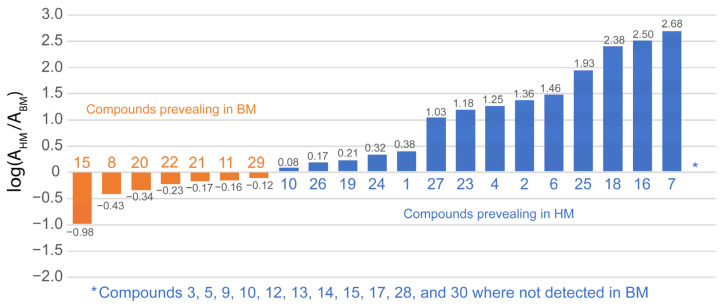
Logarithm area ratios of the identified compounds in the ethanolic extracts of HM and BM dried walnut shells. Compounds in orange (blue) denote higher contents in the BM (HM) samples. Several hydrolyzable tannins and ellagic acid derivatives reported in Table 2 were not detected in the BM and were not reported in the graph.

To understand and emphasize the differences between HM and BM walnut shell extract samples, Figure 7 presents the logarithm of the ratio between the area of a specific compound in the HM and BM samples. This graphical representation allows for a direct assessment of the impact of the grinding process. A negative value implies a higher abundance of the compound in the BM walnut shell sample, while positive values suggest the opposite. Values close to zero indicate minimal differences between the two samples. The observations regarding the BM process are indeed fascinating, as they demonstrate, for example, an exponential decrease in the concentrations of hydrolyzable tannins. In particular, the concentrations of monogalloyl-hexose isomers show a significant decrease by 1–2 orders of magnitude. What is even more remarkable is that the compounds containing the hexahydroxydiphenoyl (HDDP) moiety are depleted, leaving no trace in the BM sample. Thus, they are missing in Figure 7.

In contrast, the compounds containing quercetin (including hexoside and pentoside forms) seem to be more abundant in the BM-extracted sample. This suggests that these compounds exhibit greater resistance to the grinding process and potentially undergo a more effective extraction. Notably, the higher concentration of benzoic acid in the BM sample exceeds that of the HM sample by about one order of magnitude. This observation suggests that benzoic acid is a by-product formed from the hydrolysis and oxidative cleavage of larger polyphenolic compounds, which are present in the walnut shells during the ball milling process.

### 2.5. Total Phenol Content and Radical Scavenger Activity (RSA)

**Figure 8:**
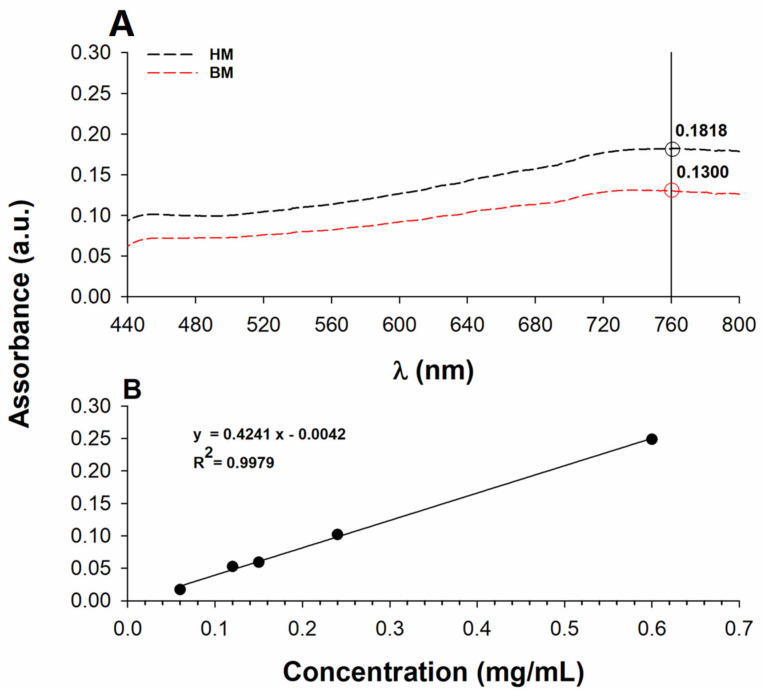
(**A**) UV–Vis spectra of the ethanolic extract of walnut shells. The TPC was established by the Folin–Ciocalteu assay, monitoring the absorbance at 760 nm. (**B**) Calibration curve of gallic acid used to establish the TPC of walnut shell extracts via the Folin–Ciocalteu method.

The molecular investigation of phenol components revealed significant changes between HM and BM processes that affect the total RSA. However, the distribution of species in various phenol classes may have a minor impact on the total polyphenol content (TPC) of walnut shell extracts. The Folin–Ciocalteu assay revealed that the HM extract exhibited a slightly higher phenol content compared to the BM one (Figure 8A). Absorption values for the diluted walnut shell samples fell within the linear range of the calibration curve, as shown in Figure 8B.

Considering the dilution factor, the antioxidant contents in terms of gallic acid equivalent were 88 ± 4 μg/mL and 63 ± 4 μg/mL in the HM and BM samples, respectively. The antioxidant content as absolute values were 35.1 ± 1.6 mg/(g sample) and 25.1 ± 1.5 mg/(g sample). These values reveal that the walnut shell extracts contain a high level of antioxidant compounds, supporting the idea that this by-product should be regarded as a resource rather than waste. It is worthwhile mentioning that these results are consistent with previous studies [49], showing a good correlation between the TPC and antioxidant capacity in chestnut seeds and/or shells. The high TPC in walnut shells likely contributes to their antioxidant activity, possibly due to the presence of phenolic and unsaturated species.

## 3. Materials and Methods

### 3.1. Chemicals

LC-MS grade water, acetonitrile, methanol (MeOH), hexane, chloroform (CHCl_3_, HPLC grade), gallic acid, ammonium acetate (reagent grade), Folin–Ciocalteu solution, sodium carbonate (Na_2_CO_3_), disodium hydrogen phosphate, sodium dihydrogen phosphate, *meta*-chloroperoxybenzoic acid (*m*-CPBA), *Candida rugosa* lipase (labelled as CRL, L1754 Type VII, ≥700 unit/mg solid) were purchased from Merck (Milan, Italy). Absolute ethanol (EtOH) was obtained from Panreact (99.8% *v*/*v*).

### 3.2. Ethanol Extraction of Dried Walnut Shells

Walnuts were cultivated in Apulia (Italy) and gently provided by a local farmer. Powders of walnut shells were obtained using a procedure reported in the literature [12]. Dried walnut shells were crushed into small fragments using a hammer and sieved with a 1 mm sieve (sample HM). The dried fragments of walnut shell, each smaller than 1 mm in size due to *hammer milling*, were further reduced in size by means of a planetary ball miller (Fritsch Pulverisette 7 planetary micro mill) equipped with two 12 mL capacity stainless steel reactors. Each reactor was filled with approximately 2 g of dried biomass (*d*/*w*). Additionally, 10 stainless steel spheres with a diameter of 5 mm and 3 stainless steel spheres with a diameter of 7 mm were included within each reactor. The milling protocol consisted of 8 cycles, each lasting 5 min at a rotation speed of 450 rpm. Following each cycle, a 10-min pause was implemented to prevent biomass heat build-up, thus avoiding putative structural modifications of extracted compounds. Following the completion of milling cycles, the temperature remained below 50 °C.

The resulting biomass was sieved with a 0.5 mm sieve. HM and BM samples were then subjected to Soxhlet extraction [50] for 12 h using EtOH, during which the temperature approached the boiling point of ethanol (78 °C).

### 3.3. Sample Preparation

For lipid extraction, 10 mg of walnut shell extract was dissolved in 2 mL of MeOH and left in an ultrasonic bath at 60 °C for 5 min, after which the organic phase was recovered, and 2 aliquots of 100 µL of the mixture were brought to dryness under N_2_. Enzymatic digestion was carried out on an aliquot of ethanolic extract following a previous protocol [29]. The sample was dissolved in 900 µL of phosphate buffer (pH 7.9) and 300 µL of *Candida rugosa* lipase (0.25 mg/mL) suspension. The digestion was incubated at 37 °C for 45 min; subsequently, 1 mL of CHCl_3_ and 1 mL of H_2_O were added to the sample and vortexed before undergoing centrifugation for 10 min at 3000× *g*. The organic phase was collected, subjected to a gentle N_2_ drying, and reconstituted in 100 µL of the mobile phase initial composition, ready for FA analysis. To establish the positioning of double bonds in unsaturated FAs, a recently developed protocol [21] was applied. Briefly, a dried sample was dissolved in 100 µL of a CHCl_3_ suspension containing 2 mg/mL of *m*-CPBA. The epoxidation reaction occurred at room temperature for 15 min. To halt the epoxidation process, 500 µL of pure water was added. The resulting solution underwent N_2_ drying, followed by resuspension in 100 μL of the initial mobile phase composition, ready to be injected into the LC-ESI-MS system. Triplicate measurements were carried out, and average values were computed. The characterization of phenolic compounds was carried out with approximately 1.0 mg of sample dissolved in 1 mL of MeOH, sonicated at room temperature for 10 min. Upon sample filtration, it was injected into the LC-MS system.

### 3.4. Attenuated Total Reflection (ATR) Fourier-Transformed Infrared Spectroscopy (FTIR)

ATR-FTIR spectra were recorded on a Spectrum Two spectrophotometer (Perkin Elmer, Waltham, MA, USA) with a DTGS detector in the region of 4000–600 cm^−1^ at a resolution of 0.5 cm^−1^, using a scan time of 2 min with 15 scanning transients.

### 3.5. RPLC-ESI-MS Instrumentation and Operating Conditions

An Ultimate 3000 UHPLC chromatographic system coupled with a heated electrospray source ionization (ESI) and a Q-Exactive quadrupole-Orbitrap^TM^ mass spectrometer (Thermo Scientific, Waltham, MA, USA) was employed.

For FA analysis, an Ascentis Express C18 column packed with core-shell particles (150 × 2.1 mm ID, 2.7 µm particle size, 1.7 µm core size), equipped with an Ascentis Express C18 (5 × 2.1 mm ID) security guard cartridge (Supelco, Bellefonte, PA, USA), at a temperature of 40 °C was used. The binary gradient elution reported in [20] and flow rate of 200 µL/min were applied.

For phenol analysis, the same apparatus was employed at 25 °C, using a flow rate of 200 µL/min. RPLC separations were performed using a binary gradient based on water (eluent A) and acetonitrile (60/40, *v*/*v*) (eluent B), both containing 0.1% formic acid. The gradient elution program was the following: 0–5 min isocratic at 5% (*v*/*v*) solvent B; 5–10 min linear from 5% to 50% (*v*/*v*) solvent B; 10–20 min linear from 50% to 100% (*v*/*v*) solvent B; 20–30 min isocratic at 100% solvent B; 30– 32 min linear from 100% to 5% (*v*/*v*) solvent B, followed by 10 min equilibration time.

The Q-Exactive heated ESI interface (Thermo Scientific, Waltham, MA, USA) and ion optics parameters were configured as follows: sheath gas flow rate at 35 arbitrary units (a.u.); auxiliary gas flow rate at 15 a.u.; spray voltage at −2.5 kV (negative polarity); capillary temperature maintained at 320 °C; and S-lens radio frequency level set at 100 a.u. Full scan MS acquisitions were performed in negative ion mode within the *m*/*z* range of 100–1000, employing a mass resolving power of 70,000 (at *m*/*z* 200). During MS measurements, the Orbitrap fill time was set to 200 ms, and the automatic gain control (AGC) level was set to 3 × 10^6^. MS/MS acquisitions were performed in negative-ion mode on targeted precursor ions using an isolation window of 1 *m*/*z* centred on it, a resolving power of 17,500 (at *m*/*z* 200), a fill time of 100 ms, and AGC of 2 × 10^5^; normalized collision energy (NCE) was typically 35%.

The LC-MS management and initial data processing were effected using the Xcalibur software 2.2 SP1.48 (Thermo Scientific, Waltham, MA, USA). MS raw data were imported, elaborated, and finally turned into figures by SigmaPlot 14 software (Systat Software, Inc., London, UK).

### 3.6. Determination of Total Polyphenol Content (TPC) by Folin–Ciocalteu Assay

The TPC was evaluated by the Folin–Ciocalteu method given by Dewanto et al. with some adjustments [51]. Briefly, 5 µL of extract solution (2.5 mg/mL in MeOH/H_2_O 80:20, *v*/*v*) was mixed with 120 µL of distilled H_2_O and 125 µL of Folin–Ciocalteu solution, stirred, and left to rest for 6 min. Then, 1.25 mL of 7% Na_2_CO_3_ was added, and the obtained mixture was held in the dark for 1.5 h at room temperature. Blank samples were prepared in the same way by using distilled H_2_O. Upon incubation, the absorbance was measured spectrophotometrically at 760 nm using a double-beam spectrophotometer Shimadzu UV-1601 UV-Vis (Shimadzu Corporation, Kyoto, Japan). A gallic acid calibration curve (60–600 mg/mL) was used to determine the TPC of the extracts. Three measurements were used to calculate the average data.

## 4. Conclusions

This study provides valuable insights into the composition of fatty acids and phenolic compounds occurring in the ethanolic extracts of dried walnut shells obtained through two different preparation methods, i.e., HM and BM pre-treatments. The ATR-FTIR analysis offered a qualitative evaluation into the overall composition differences. The LC-MS analysis of the enzymatic hydrolyzate samples revealed similar FA distributions in both HM and BM extracts with the following most abundant species, *viz.* FA 18:2 Δ^9,12^, FA 18:1 Δ^9^, and FA 18:3 Δ^9,12,15^. On the contrary, important differences were observed in the composition of phenolic compounds. The HM sample showed a significant presence of ellagitannins and hydrolyzable tannins, which were nearly absent in the BM one. This difference might be responsible for the change in antioxidant activity observed between the two ethanolic extracts of walnut shells. Both samples exhibited numerous bioactive components, including phenols, ellagic acid derivatives, flavonoids, and ellagitannins, which corroborate the phenolic content identified through the Folin–Ciocalteu test.

Overall, these findings underscore the importance of extraction methods in shaping the composition of bioactive metabolites. The study also emphasizes the value of sustainable and efficient approaches to utilize agricultural waste for beneficial purposes. Looking towards the future, we can gain valuable insights into the significance of walnut biomass pre-processing by exploring different walnut cultivars from various geographical regions and examining other waste components like hulls.

## Figures and Tables

**Table 1:** List of saturated and unsaturated (mono-, di-, or triply unsaturated) fatty acids identified in the dried hard shells of walnut (*Juglans regia* L.) samples.

N.	FA ^a^	RT (min)	[M-H]^−^ *m*/*z*	Relative Abundance (%)		epo-FA	RT (min)	[epoM-H]^−^ *m*/*z*	DB Location ^b^	Diagnostic Ions (*m*/*z*)
				**HM**	**BM**					
1	14:0	35.4	227.2017	0.49	0.55	**-**	**-**	-	-	-
2	15:0	39.0	241.2173	0.39	0.45	-	-	-	-	-
3	16:0	41.1	255.2330	14.27	15.65	-	-	-	-	-
**4**	**16:1**	**37.6**	**253.2173**	**0.67**	**0.76**	**epo-FA 16:1**	**31.0**	**269.2111**	**Δ^9^**	**155.108/171.103**
5	17:0	44.7	269.2486	0.87	1.17	-	-	-	-	-
**6**	**18:0**	**47.0**	**283.2643**	**9.83**	**10.49**	**-**	**-**	**-**	**-**	**-**
**7**	**18:1**	**43.5**	**281.2486**	**18.30**	**16.84**	**epo-FA 18:1**	35.9 **36.5**	**297.2424**	Δ^11^ **Δ^9^**	183.139/199.134 **155.108/171.103**
**8**	**18:2**	**40.1**	**279.2330**	**42.38**	**40.96**	**epo^2^-FA 18:2**	**21.2**	**311.2216**	**Δ^9, 12^**	**155.108/171.103** **211.134/227.129** **193.123/209.118**
									Δ^11, 14^	183.139/199.134
**9**	**18:3**	**36.7**	**277.2173**	**9.60**	**9.90**	**epo^3^-FA 18:3**	**4.50**	**325.2009**	**Δ^9, 12, 15^**	**155.108/171.103** **211.134/227.129** **193.123/209.118** **249.149, 267.160**
10	19:0	49.1	297.2799	0.17	0.16	-	-	-	-	-
11	20:0	50.8	311.2956	0.50	0.51	-	-	-	-	-
12	20:1	47.9	309.2799	0.16	0.17	epo-FA 20:1	41.5	325.2009	Δ^11^	183.139/199.134
13	21:0	52.4	325.3112	0.20	0.23	-	-	-	-	-
14	22:0	53.8	339.3269	0.58	0.61	-	-	-	-	-
15	23:0	55.0	353.3425	0.57	0.58	-	-	-	-	-
16	24:0	56.2	367.3582	1.02	0.97	-	-	-	-	-

^a^ Fatty acids (FAs) are indicated with C carbon atoms in the side chain and DB number of C-C double bonds after a colon (C:DB); the most abundant detected species are reported in bold. ^b^ Δ is the position of the first double bond counted from the carboxylic moiety.

**Table 2:** Phenolic compounds identified in the ethanolic extracts of dried walnut (*Juglans regia* L.) shell samples.

n	*m*/*z* [M-H]^−^	RT	Formula	MS/MS Fragments	Putative Identification ^a^	Classification	HM	BM
1	191.0561	1.5	C_7_H_12_O_6_	191.056 (100)–85.029 (10)–173.040 (5)	Quinic acid	Hydrolyzable Tannins	0.18	0.43
2	331.0671	1.5	C_13_H_16_O_10_	169.014 (100)–211.025 (30)–271.046 (25)	Monogalloyl-glucose (1)	Hydrolyzable Tannins	0.05	1.02
3	481.0624	1.5	C_20_H_18_O_14_	300.999 (100)–275.020 (60)–249.040 (10)	HHDP-glucose (1)	Hydrolyzable Tannins	n.d.	4.63
4	331.0671	2.0	C_13_H_16_O_10_	169.014 (100)–211.025 (50)–271.046 (20)	Monogalloyl-glucose (2)	Hydrolyzable Tannins	0.03	0.6
5	481.0624	2.0	C_20_H_18_O_14_	300.999 (100)–275.020 (60)–249.040 (10)	HHDP-glucose (2)	Hydrolyzable Tannins	n.d.	2.8
6	169.0143	2.2	C_7_H_6_O_5_	125.024 (100)–169.0142 (40)	Gallic Acid	Phenolic acid	0.1	2.93
7	331.0671	2.3	C_13_H_16_O_10_	169.014 (100)–125.024 (15)–271.046 (2)	Monogalloyl-glucose (3)	Hydrolyzable Tannins	n.d.	0.26
8	181.0507	3.4	C_9_H_10_O_4_	151.040 (100)–123.045 (50)	Syringaldehyde	Phenolic acid	13.13	4.76
9	783.0687 391.0312 ^b^	3.9	C_34_H_24_O_22_	300.999 (100)–275.020 (45)–481.063 (5)	Bis-HHDP-glucose (1)	Hydrolyzable Tannins	n.d.	4.36
10	153.0194	4.5	C_7_H_6_O_4_	109.029 (100)–153.019 (40)	Protocatechuic acid	Phenolic acid	1.91	2.22
11	137.0244	8.0	C_7_H_6_O_3_	137.023 (100)–93.035 (10)	Hydroxybenzoic acid	Phenolic acid	8.37	5.6
12	783.0687 391.0312 ^b^	10.9	C_34_H_24_O_22_	300.999 (100)–275.020 (45)–481.063 (5)	Bis-HHDP-glucose (2)	Hydrolyzable Tannins	n.d.	7.28
13	633.0734	12.2	C_27_H_22_O_18_	300.999 (100)–275.020 (15)–463.0525 (10)	Galloyl-HHDP- glucose	Hydrolyzable Tannins	n.d.	1.14
14	785.0843 392.0392 ^b^	12.3	C_34_H_26_O_22_	300.999 (100)–275.020 (45)–249.040 (30)	Digalloyl-HHDP- glucose	Hydrolyzable Tannins	n.d.	1.54
15	121.0295	12.6	C_7_H_6_O_2_	121.029 (100)	Benzoic acid	Phenolic acid	58.33	5.89
16	463.0518	12.7	C_20_H_16_O_13_	299.992 (100)–300.999 (80)	Ellagic acid hexoside	Ellagic Acid derivatives	n.d.	0.66
17	785.0843 392.0392 ^b^	13.0	C_34_H_26_O_22_	300.999 (100)–275.020 (45)–249.040 (30)	Digalloyl-HHDP- glucose	Hydrolyzable Tannins	n.d.	4.07
18	433.0412	13.1	C_19_H_14_O_12_	299.992 (100)–300.999 (80)	Ellagic acid pentoside	Ellagic Acid derivatives	0.01	2.55
19	391.1035	13.4	C_19_H_20_O_9_	137.024 (100)–281.067 (90)–109.029 (20)	Lanceoloside A isomer	Benzoylglucoside	9.98	4.38
20	405.1192	13.4	C_20_H_22_O_9_	137.024 (100)–281.067 (90)–123.040 (80)	Benzoyl derivate of Lanceoloside A isomer	Benzoylglucoside	4.07	2.69
21	197.0455	13.5	C_9_H_10_O_5_	167.035 (100)–139.039 (20)–182.021 (12)	Syringic acid	Phenolic acid	0.09	0.05
22	197.0455	13.5	C_9_H_10_O_5_	197.046 (100)–169.014 (35)–125.024 (12)	Ethyl gallate	Phenolic acid	0.4	5.87
23	463.0883	13.5	C_21_H_20_O_12_	300.028 (100)–301.035 (50)–161.043 (10)	Quercetin glucoside	Flavonoid	0.14	0.28
24	300.9990	13.6	C_14_H_6_O_8_	300.999 (100)–283.995 (1)–257.010 (1)	Ellagic Acid	Ellagic Acid derivatives	0.1	8.37
25	433.0780	13.7	C_20_H_18_O_11_	300.028 (100)–301.035 (50)	Quercetin pentoside	Flavonoid	0.12	0.18
26	447.0935	13.8	C_21_H_20_O_11_	300.028 (100)–301.035 (80)–271.024 (10)	Quercitrin	Flavonoid	1.89	2.7
27	181.0506	14.6	C_9_H_10_O_4_	181.051 (100)–151.019 (40)–109.029 (12)	Ethyl protocatechuate	Phenolic acid	0.19	1.91
28	937.0953 468.0440 ^b^	15.4	C_41_H_30_O_26_	169.014 (100)–300.999 (75)–275.020 (25)	Drigalloil-HHDP- glucose	Hydrolyzable Tannins	n.d.	5.35
29	271.0613	15.7	C_15_H_12_O_5_	151.004 (100)–119.004 (40)–93.034 (20)	Naringenin	Flavanone	0.91	0.67
30	935.0796 467.0365 ^b^	16.5	C_41_H_28_O_26_	300.999 (100)–275.020 (20)–249.040 (5)	Digalloyl-bis-HHDP-glucose	Hydrolyzable tannins	n.d.	14.82

^a^ Recognitions based on accurate *m*/*z* values and fragmentation pattern; the most abundant species are reported in bold. ^b^ Bisdeprotonated ion [M-2H]^2−^.

## Data Availability

Data will be available upon request.
